# Effect of Sevoflurane, Isoflurane, and Desflurane on the Random Blood Glucose Levels in Non-diabetic Patients Undergoing General Anesthesia: A Randomized, Single-Blind Study

**DOI:** 10.7759/cureus.54216

**Published:** 2024-02-14

**Authors:** Chandini Kukanti, Nandkishore Agrawal, Habib Md R Karim, Mamta Sinha

**Affiliations:** 1 Anaesthesiology, Critical Care, and Pain Medicine, All India Institute of Medical Sciences, Raipur, Raipur, IND; 2 Anaesthesiology, Critical Care, and Pain Medicine, All India Institute of Medical Sciences, Deoghar, Deoghar, IND

**Keywords:** stress hyperglycemia, blood sugar fluctuation, volatile anesthetics, anesthesia complications, perioperative anesthesia service

## Abstract

Background: Volatile anesthetic agents like sevoflurane, isoflurane, and desflurane are widely used for maintaining general anesthesia (GA). Their effect on the autonomic system is different and can impact the blood sugar homeostasis. This study compares the intraoperative blood glucose levels in non-diabetic patients undergoing non-cardiac surgery under GA with the three volatile agents.

Methods: A randomized, single-blind, parallel-arm study recruited 105 non-diabetic patients into three groups. GA induction and maintenance were standardized except for the volatile agent. Capillary blood sugar levels were measured at different time points and compared between and within the groups. A p-value of <0.05 was considered significant.

Results: Entire participants completed the study, and their baseline characteristics were statistically indifferent. Intraoperative blood glucose rise and variation were the highest in the desflurane group and the lowest in the isoflurane group; the differences were statistically significant at 15, 30, and 45 minutes. The highest blood sugar level was noted at 60 minutes in all groups; after that, the level started falling. However, none of the raises were beyond 140 mg% to categorize them as hyperglycemia.

Conclusion: Intraoperative glycemic variation was evident with isoflurane, sevoflurane, and desflurane. The maximum increase from the pre-induction level was noted at 60 minutes. However, none of the readings reached the hyperglycemia level. The rise was significantly higher in desflurane-based anesthesia than in isoflurane. This study was, however, conducted in non-diabetic patients; hence, results might not be extrapolated to diabetic patients.

## Introduction

General anesthesia (GA) is one of the most used methods of anesthesia in rendering a patient to a state of controlled unconsciousness. Many anesthetic agents achieve this state by interfering with the functioning of the corticothalamic networks. Volatile inhalational agents are one of the most used agents for the induction and maintenance of GA. In recent days, the most used inhalational agents have been sevoflurane, isoflurane, and desflurane. Surgery evokes a series of neurohormonal and metabolic responses commonly called the stress response, i.e., a state of hypermetabolism and hypercatabolism [1]. The surgical stress response contributes to increased intraoperative blood glucose levels by activating the neuro-endocrine-metabolic and inflammatory-immune responses. Volatile anesthetic agents that maintain balanced anesthesia can interfere with the surgical stress response by decreasing the secretion of cortisol, adrenocorticotrophin, catecholamine, and growth hormone [1]. Stress also promotes insulin resistance, intraoperatively causing deranged blood glucose levels [2]. The development of insulin resistance is the primary source of a series of reactions in response to injury and the consequent metabolic state [3,4]. It can lead to adverse perioperative outcomes such as surgical site infections and prolonged hospital admissions [5].

Although anesthesia can suppress the autonomic responses, such suppression happens at a higher dose than required for maintaining surgical anesthesia levels. Therefore, knowing the impacts of commonly used volatile agents on blood glucose levels can be very beneficial in the meticulous planning of GA and thereby help reduce perioperative hyperglycemia, surgical site infections, and related morbidities.

The study result was presented at the Chhattisgarh State Conference of the Indian Society of Anaesthesiologists 2023, held at Durg, India, on September 24, 2023, and the Indian National Conference of the Indian Society of Anaesthesiologists 2023 under the Dr. TN Jha Memorial and Dr. KP Chansoriya Medal and Traveling Grant Award held at Gurgaon, India, on November 24, 2023.

## Materials and methods

Study design and settings

The present randomized, single-blind, parallel-arm study recruited humans from December 2021 to December 2022 and was conducted at All India Institute of Medical Sciences, Raipur, in Raipur, India. The project was a post-graduate thesis; the Post-Graduate Thesis Review Committee reviewed the protocol which was finally evaluated and approved by the Institute Ethics Committee of All India Institute of Medical Sciences, Raipur (approval number: AIIMSRPR/IEC/2021/960). Further, the study was registered in the Clinical Trial Registry-India (CTRI/2021/12/038579). The study follows the Declaration of Helsinki and Good Clinical Practice guidelines and is reported per the Consolidated Standards of Reporting Trials guidelines.

Participants

With informed and written consent, patients undergoing elective surgeries under GA using endotracheal intubation were included in the study. The age group of the participants was 18-60 years, and males and females belonging to the American Society of Anesthesiologists (ASA)-physical status I or II and who were non-diabetic were included. Patients who were unwilling to participate, with uncontrolled hypertensives, on drugs altering blood glucose levels like steroids, in whom inhalational anesthetic agents are contraindicated, requiring rapid sequence intubation, with pancreatic disorders, with pancreatic surgeries or with catecholamine-secreting tumors, and with surgical duration less than two hours were excluded from the study.

Sample size

The sample size was calculated based on the standard deviation (SD) and minimum difference detected for the mean difference of blood glucose at three hours baseline between sevoflurane and desflurane taken from a previous study by Haldar et al. [5]. The following formula was used, N=(2*(Zα+Zβ)^2^*σ^2 ^)/δ^2^, where N is the sample size for each group, Zα equals 1.96 at the 95% confidence interval, Zβ equals 0.84 at 80% power, σ is the combined (average) SD (8.67+21.03/2=14.85), and δ is the difference between mean (12.87-5.83=7.04). Substituting the values in the formula, we got a sample size of 35. We considered the design effect of 1 and planned allocation of 1:1:1 for three groups. It gave us a final total sample of 105 participants.

Randomization

One hundred five random numbers ranging from 1 to 105 were computer generated and stored with the investigators. Participants were allocated into the above three groups per their chosen random numbers. Allocation concealment was done by storing the group allocation data only with the primary investigator. Only the participants were blinded to the intervention; anesthesiologists and data collectors could not be blinded due to the inherent characteristics of the interventions.

Technique

On visiting the patient the night before surgery, they were advised for nil per os for six hours, tablet ranitidine 150 mg, and tablet alprazolam 0.5 mg; intravenous fluid to be given is 0.9% sodium chloride. On the day of surgery, the patients were informed about the use of volatile anesthetics to provide GA during the surgical procedure. The standard institutional practice and advantages and disadvantages of volatile anesthetics were explained to the patient, and informed consent was obtained. Only consented patients were randomized for group allocation. The patient was shifted to the operating room. On the operating table, injection of midazolam 0.03 mg/kg intravenously, injection of glycopyrrolate 0.2mg intravenously, and injection of fentanyl 2 mcg/kg were administered. After five minutes of administering this premedication, the first blood glucose level, i.e., pre-induction value, was measured. A capillary blood sample was collected under aseptic precautions from the fingertip of the arm, free of intravenous access. Blood glucose levels were measured for all patients using the OneTouch Select Plus Simple® Glucometer (LifeScan IP Holdings, LLC, Milpitas, CA).

At the same time, heart rate (HR), mean arterial pressure (MAP), and peripheral oxygen saturation (SpO_2_) were also recorded. All the patients were induced with an injection of propofol 2 mg/kg intravenously. After induction, the chosen anesthetic gas (sevoflurane/isoflurane/desflurane) was turned on, and a muscle relaxant (Inj. vecuronium 0.1 mg/kg) was administered. Intermittent positive pressure ventilation was maintained with a closed circuit. A low-flow anesthesia technique was employed in all cases. An age-adjusted minimum alveolar concentration (MACage) of 1.0±0.1 was targeted and changed later according to the patient's hemodynamic stability if required. Oxygen, nitrous oxide, the chosen anesthetic gas (sevoflurane/isoflurane/desflurane), and Inj. vecuronium 0.02 mg/kg intravenous top-ups (maximum 1 mg) were used to maintain a state of balanced anesthesia.

Readings were noted at pre-decided time points during the anesthesia maintenance phase. Finally, at the end of the surgery, the inhalational agent was turned off after skin suturing, and one more value of blood glucose level was taken after achieving MACage 0. Upon completion of the surgery, neuromuscular blockade was reversed with the injection of neostigmine 50 mcg/kg and injection of glycopyrrolate 10 mcg/kg intravenously. Then, the patient was extubated and shifted to the post-anesthesia care unit (PACU) for monitoring per the standard prevailing practice. One more reading was noted in the PACU 30 minutes after surgery. Postoperative pain was managed using the multimodal analgesia technique, and pain level was targeted below a score of 4 out of 10 on the numerical rating scale.

Outcome variables

Preoperative demographic-related data, vitals and basic hemodynamic parameters, and blood sugar levels were collected. Intraoperative blood glucose monitoring was started after obtaining MACage 1. The first value was noted after getting MACage 1 and then at every 15 minutes for the first hour, followed by every 30 minutes afterward. HR, MAP, and SpO_2_ were measured simultaneously as blood glucose levels. 

Data management and statistical analysis

Statistical analysis was carried out using statistical packages for SPSS 20 for Windows (SPSS Inc., Chicago, IL). All continuous variables were tested for normality by the Shapiro-Wilk test of normality. Normally distributed data were represented by mean±SD, while non-parametric data were represented by median (IQR). Categorical variables are expressed as absolute numbers and percentages. A one-way ANOVA test was applied to compare the three groups. Repeated measures ANOVA was used to compare at each time interval. Two-sided p-values are considered statistically significant at p<0.05. 

## Results

All participants recruited in the study completed the procedure, and data were collected and analyzed. The study population comprised 57.1% male and 42.9% female patients. The median age of the patients was 31.00 years, with a median body mass index (BMI) of 21.60 kg/m^2^. The baseline basic hemodynamic parameters, oxygen saturation, and mean level of the random blood sugar (RBS) in these patients were statistically indifferent, as shown in Table 1.

**Table 1:** The baseline demographic and vital parameters in the different groups ASA-PS: American Society of Anesthesiologists physical status; BMI: body mass index; MAP: mean arterial pressure; PR: pulse rate; RBS: random blood sugar; SpO_2_: peripheral oxygen saturation

Parameters	Groups			P-value
	Desflurane	Isoflurane	Sevoflurane	
Age in years	27.00 (38-22)	31.00 (44-27)	37.00 (42-24)	0.054
Male (n(%))	21 (60%)	23 (65.71%)	16 (45.71%)	0.219
Female (n(%))	14 (40%)	12 (34.29%)	19 (54.29%)	0.219
BMI kg/m^2^	20.7 (22.3-19.4)	22.4 (24.3-20.1)	21.6 (23.4-20.2)	0.088
PR beats/min	77.71±10.68	79.94±14.01	76.91±12.02	0.569
MAP in mmHg	83.94±6.98	84.41±8.99	83.51±8.11	0.593
SpO_2 _(%)	100 (100-100)	100 (100-100)	100 (100-100)	0.114
ASA-PS I (n(%))	28 (80%)	26 (74.29%)	28 (80%)	0.8004
ASA-PS II (n(%))	7 (20%)	9 (2.71%)	7 (20%)	0.8004
RBS in mg%	92.97±5.24	89.43±9.45	92.17±8.27	0.146

There was no statistical significance in the intraoperative variables after achieving MACage 1. The median pulse rate, MAP, and SpO_2_ did not show any significant difference between the three groups at any interval. The capillary blood glucose was the highest in the patients being anesthetized by desflurane. In contrast, the patient group who were administered isoflurane showed the slightest increase in the RBS, and it was statistically significant at 15 minutes (p-value: 0.042), 30 minutes (p-value: 0.006), and 45 minutes (p-value: 0.039). The within-group difference was statistically significant (Table 2).

**Table 2:** The mean values of random blood sugar from pre induction through intraoperative to postoperative in the three groups MACage: age-adjusted minimum alveolar concentration

Time train	Anesthetics			P-value
	Desflurane	Isoflurane	Sevoflurane	
Pre induction (PI)	89.43±8.12	89.00±9.34	90.11±9.23	0.872
Intraoperative				
MACage 1	92.43±12.06	89.60±10.50	90.37±10.46	0.753
15 mins	100.60±13.42	92.86±12.18	94.74±13.99	0.042
30 mins	106.69±16.87	96.37±11.27	97.63±14.64	0.006
45 mins	109.80±16.30	101.34±12.64	102.57±15.07	0.039
60 mins	110.20±15.29	103.00±15.29	105.34±19.31	0.169
90 mins	109.09±16.69	101.94±12.18	104.97±18.70	0.181
120 mins	107.40±15.22	101.51±12.54	102.23±15.67	0.187
MACage 0	107.69±12.40	101.89±11.60	101.60±14.31	0.086
Average intraoperative	105.48±13.36	98.56±8.89	99.93±13.31	0.043
Postoperative	106.69±11.63	101.83±11.49	101.17±16.98	0.185
P-value (PI – mean intraoperative)	<0.001	<0.001	<0.001	

Table 3 gives an intergroup comparison and their p-values. The p-values were significant between the desflurane and isoflurane groups at the 15-minute, 30-minute, and 45-minute time intervals and desflurane and sevoflurane at only the 30-minute time point. There was no significant difference in the mean RBS levels between the isoflurane and sevoflurane groups.

**Table 3:** The intergroup comparison of random blood sugar levels MACage: age-adjusted minimum alveolar concentration

Time train	Intergroup – p-value		
	Desflurane/isoflurane	Isoflurane/sevoflurane	Sevoflurane/desflurane
Pre induction	0.963	0.860	0.962
MACage 1	0.736	0.956	0.890
15 mins	0.042	0.822	0.158
30 mins	0.010	0.930	0.027
45 mins	0.048	0.935	0.106
60 mins	0.155	0.817	0.423
90 mins	0.157	0.712	0.535
120 mins	0.213	0.977	0.301
MACage 0	0.146	0.995	0.121
Average intraoperative	0.043	0.883	0.136
Postoperative	0.298	0.978	0.212

**Figure 1:**
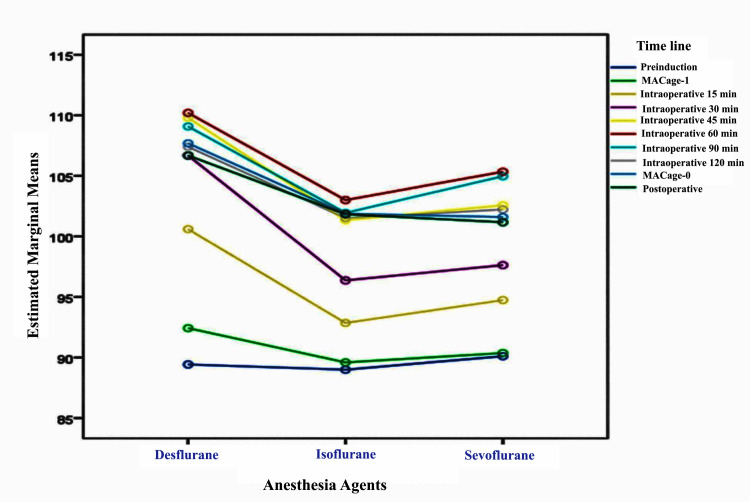
Time trend lines of random blood sugar for the three groups

The highest rise in the intraoperative RBS was seen in the 60 minutes for all the agents. The rise was the highest in the desflurane group and the lowest in the isoflurane group. The time trend lines of RBS for the three groups are presented in Figure 1.

A paired t-test was done to compare the pre-induction RBS level with the average intraoperative RBS levels. There was a significant increase in the RBS in all the patients. The mean difference between the pre induction and intraoperative was the highest in the patient group administered with desflurane and the lowest in patients anesthetized with isoflurane (Table 4).

**Table 4:** Paired t-test of pre induction against intraoperative random blood sugar levels SD: standard deviation

Anesthetic agents	Pre induction		Average intraoperative		Paired difference		T	P
	Mean	SD	Mean	SD	Mean	SD		
Desflurane	89.26	8.18	106.18	12.90	-16.91	13.47	-7.320	<0.001
Isoflurane	89.00	9.34	98.56	8.89	-9.56	11.96	-4.728	<0.001
Sevoflurane	90.11	9.27	99.93	13.32	-9.82	11.47	-5.065	<0.001
Total	89.46	8.881	101.51	12.21	-12.05	12.67	-9.704	<0.001

## Discussion

The present study evaluated three commonly used modern-day volatile anesthetic agents, sevoflurane, isoflurane, and desflurane, and found that all agents tested could maintain euglycemia during the intraoperative and immediate postoperative period. However, there was a significant variation in the glycemia level within and among the groups where the desflurane-based anesthesia showed higher blood sugar levels. Perioperative glycemic control plays a significant role in improving surgical outcomes and decreasing morbidity both in patients with or without diabetes mellitus and even in non-cardiac surgeries [6,7]. Nevertheless, intraoperative increases in blood glucose levels and variation can be attributed to various factors [8]. Pain, depth of anesthesia, surgical duration, and drugs that alter blood sugar homeostasis are a few of them. In the present study, the anesthesia induction, maintenance, and reversal were standardized. We did not enroll patients on drugs that could affect their blood sugar levels or did not administer any such drugs during the study duration. Further, we kept the data collection fixed to the two hours of anesthesia, to make it homogenous. 

The mechanism can be attributed to the inherent property of desflurane on the sympathetic nervous system. The sympathetic nervous system plays a significant role in maintaining homeostasis by regulating the insulin level. When activated, the sympathetic system releases norepinephrine, which inhibits insulin release from the pancreas, thereby reducing glucose uptake in peripheral tissues and increasing glucose output from the liver. Several factors that can increase the sympathetic tone include pain and psychological or surgical stress. Desflurane has been shown to increase the sympathetic system's activity, especially when the concentration administered changes suddenly. One hypothesis is that desflurane causes desensitization of the baroreceptor reflexes [9]. The increased sympathetic tone can alter the circulating insulin levels and affect insulin sensitivity, leading to increased blood glucose levels.

The concept of suppressing autonomic response to the surgical stimulus is usually represented by MAC-bar [10]. However, the concentration of volatile anesthetic required for MAC-bar is usually very high, which can significantly impact the hemodynamics. Further, the current standard practice of balanced anesthesia does not use such a high concentration. Nevertheless, even the concentrations used for balanced anesthesia have also been investigated to evaluate the effect on perioperative glucose homeostasis and fund varied results.

Notably, the intraoperative blood sugar variation during volatile anesthesia can not only be attributed to desflurane's ability to affect the autonomic system. Iwasaka et al. studied the effect of prolonged sevoflurane anesthesia on insulin sensitivity by administering two successive glucose tolerance tests intraoperatively in eight patients undergoing prolonged surgeries [11]. It was found that the total insulin output was higher after the first glucose tolerance test compared with the second (1.161±830 vs. 568±389 pU.min.ml^-1^, p-value<0.05), thereby indicating that glucose tolerance is diminished in patients undergoing prolonged sevoflurane anesthesia [11]. Tanaka et al. studied the dose-dependent effects of sevoflurane and isoflurane anesthesia on human glucose tolerance. A glucose tolerance test was performed before the skin incision; plasma glucose and insulin levels were measured regularly. The difference in blood glucose and insulin levels was not statistically significant between the sevoflurane and isoflurane groups up to MACage 1.5 [12].

Desflurane's effect has been compared earlier with sevoflurane and propofol [5,13]. Kaushal et al. studied the effect of sevoflurane versus desflurane on blood glucose levels in patients undergoing intracranial neurosurgery. It was concluded that desflurane caused an initial rise followed by a decline. Sevoflurane caused a gradual increase in intraoperative blood glucose levels. The intraoperative change in blood sugar was statistically significant but was within the normal clinical range [13]. Haldar et al. compared the intraoperative blood glucose levels in patients anesthetized with sevoflurane, desflurane, and propofol. They concluded that in normoglycemic patients, maintenance of anesthesia with either an intravenous agent such as propofol or inhalational agents such as sevoflurane and desflurane causes a rise in blood sugar levels within the normal range. The rise was significantly higher with the inhalational agent group than with the propofol group. Among the desflurane and sevoflurane groups, the intraoperative blood glucose levels were higher in the desflurane group [5].

In our study, after achieving MACage 1 post induction, the blood glucose levels were checked at 15-minute, 30-minute, 45-minute, 60-minute, 1-hour, and 2-hour intervals. A steady rise in the blood glucose levels was observed in all three groups at regular intervals. All three groups saw the highest rise in blood glucose levels at the 60-minute interval. The difference in blood glucose levels between the three groups was insignificant from the 60-minute to 120-minute intervals. The highest rise in intraoperative blood glucose levels was seen with desflurane, whereas isoflurane had the slightest increase. The p-values were significant between desflurane and isoflurane groups at 15-, 30-, and 45-minute intervals. Although the increase in blood glucose levels was statistically significant at certain time intervals, this increase was not clinically significant. None of the patients in all three groups had increased blood glucose levels requiring intraoperative insulin administration. All these findings correlate with the previous findings.

Our study has a minor protocol deviation. Although we decided to take RBS reading at MACage of 0 after reversal, it was practically difficult as it would require a substantial waiting time and prolong the extubation. So, we measured the values mostly at MACage 0.1. 

Our study, however, has several limitations. The data are from a single tertiary care institute and include only patients with ASA physical statuses I and II. In contrast, candidates with a higher ASA physical status risk higher glycemic variability [8]. Although we standardized the depth of anesthesia, we did not keep the continuous data to compare them. Further, long-duration anesthesia and surgery have shown different responses among the inhalational anesthetic groups [14]. Although all patients underwent major surgeries, the impact of invasiveness on stress levels was not measured by levels of plasma hormones and catecholamines. This study estimated blood glucose levels to reflect neurohormonal status. The study was done in adult patients undergoing non-cardiac surgeries; pregnant women and patients with diabetes and other co-morbidities were excluded. This data cannot be applied to the general population, such as elderly patients, pediatrics, and neonates, whereas the effect of inhalational anesthesia might differ in adults and neonates [15].

## Conclusions

All volatile anesthetic agents compared in this study have been shown to increase intraoperative blood glucose levels. The maximum increase was noted at 60 minutes of anesthesia for all agents. A maximum increase in intraoperative blood glucose levels was seen with desflurane, and isoflurane had the most minor increase. However, this study was conducted in non-diabetic, ASA physical status I and II patients, and data collection was limited to an initial two hours during the intraoperative period. Hence, these results might not be representative of diabetic patients and prolonged anesthesia periods, which necessitates further research.

## References

[1] Cusack B, Buggy DJ (2020). Anaesthesia, analgesia, and the surgical stress response. BJA Educ.

[2] Soop M, Nygren J, Thorell A, Ljungqvist O (2007). Stress-induced insulin resistance: recent developments. Curr Opin Clin Nutr Metab Care.

[3] Thorell A, Nygren J, Ljungqvist O (1999). Insulin resistance: a marker of surgical stress. Curr Opin Clin Nutr Metab Care.

[4] Şimşek T, Şimşek HU, Cantürk NZ (2014). Response to trauma and metabolic changes: posttraumatic metabolism. Ulus Cerrahi Derg.

[5] Haldar R, Kannaujia AK, Verma R, Mondal H, Gupta D, Srivastava S, Agarwal A (2020). Randomized trial to compare plasma glucose trends in patients undergoing surgery for supratentorial gliomas under maintenance of sevoflurane, desflurane, and propofol. Asian J Neurosurg.

[6] Kwon S, Thompson R, Dellinger P, Yanez D, Farrohki E, Flum D (2013). Importance of perioperative glycemic control in general surgery: a report from the Surgical Care and Outcomes Assessment Program. Ann Surg.

[7] Duggan EW, Carlson K, Umpierrez GE (2017). Perioperative hyperglycemia management: an update. Anesthesiology.

[8] Sermkasemsin V, Rungreungvanich M, Apinyachon W, Sangasilpa I, Srichot W, Pisitsak C (2022). Incidence and risk factors of intraoperative hyperglycemia in non-diabetic patients: a prospective observational study. BMC Anesthesiol.

[9] Ebert TJ, Perez F, Uhrich TD, Deshur MA (1998). Desflurane-mediated sympathetic activation occurs in humans despite preventing hypotension and baroreceptor unloading. Anesthesiology.

[10] Eger EI 2nd (2001). Age, minimum alveolar anesthetic concentration, and minimum alveolar anesthetic concentration-awake. Anesth Analg.

[11] Iwasaka H, Itoh K, Miyakawa H, Kitano T, Taniguchi K, Honda N (1996). Glucose intolerance during prolonged sevoflurane anaesthesia. Can J Anaesth.

[12] Tanaka K, Kawano T, Tsutsumi YM (2011). Differential effects of propofol and isoflurane on glucose utilization and insulin secretion. Life Sci.

[13] Kaushal A, Bindra A, Dube SK (2022). Effect of sevoflurane versus desflurane on blood glucose level in patients undergoing intracranial neurosurgery: a randomised controlled study. Indian J Anaesth.

[14] Nishiyama T, Yamashita K, Yokoyama T (2005). Stress hormone changes in general anesthesia of long duration: isoflurane-nitrous oxide vs sevoflurane-nitrous oxide anesthesia. J Clin Anesth.

[15] Yu Q, Li J, Dai CL, Li H, Iqbal K, Liu F, Gong CX (2020). Anesthesia with sevoflurane or isoflurane induces severe hypoglycemia in neonatal mice. PLoS One.

